# Comparative assessment of a virtual performance measure with self-report and performance-based outcomes in patients with hip osteoarthritis

**DOI:** 10.1186/s12891-025-08282-y

**Published:** 2025-01-08

**Authors:** Helen Razmjou, Suzanne Denis, Susan Robarts, Amy Wainwright, Patricia Dickson, Ania Roszkowski, John Murnaghan

**Affiliations:** 1 Department of Rehabilitation, Holland Orthopaedic & Arthritic Centre, Sunnybrook Health Sciences Centre, Toronto, Canada; 2 Department of Physical Therapy, Faculty of Medicine, University of Toronto, Toronto, Canada; 3 Sunnybrook Research Institute, Sunnybrook Health Sciences Centre, Toronto, Canada; 4 Department of Occupational Therapy, Faculty of Medicine, University of Toronto, Toronto, Canada; 5 Division of Orthopedic Surgery, Department of Surgery, Sunnybrook Health Sciences Centre, Toronto, Canada; 6 Division of Orthopaedic Surgery, Department of Surgery, Faculty of Medicine, University of Toronto, Toronto, Canada; 7 Address: Holland Orthopaedic and Arthritic Centre, 43 Wellesley Street East, Toronto, ON M4Y 1H1 Canada

**Keywords:** Virtual, Hybrid, Performance-based measures, Accuracy

## Abstract

**Background:**

The purposes of this study were to examine the reliability and factorial and convergent validity of a virtual performance measure (VPM) in patients with osteoarthritis (OA) of the hip joint and to compare the known-group validity of the VPM with traditional self-report and performance-based outcomes.

**Methods:**

The VPM score was based on the results of 10 videos showing increasing difficulty in performing specific functional tasks. Patients were requested to choose the video that best reflected their own level of function. Clinical presentation and radiological findings were documented. Self-report measures were the lower extremity functional score (LEFS) and pain scale. The performance-based measures were the 30- second Chair Stand Test (CST) and the 40-meter fast paced walk test (40 m FPWT) test.

**Results:**

Data of 100 patients, 64 (64%) females, mean age: 67 ±10 were examined. The Cronbach’s alpha coefficient that examined internal consistency of the VPM total score was 0.88. Factor analysis showed two distinct domains. Moderate correlations were observed between the VPM total score and the LEFS, pain score, and 40 m FPWT (*r* > 0.50). The VPM and the LEFS were able to differentiate between candidates and non-candidates for hip arthroplasty and between those with and without assistive walking devices. There was no statistically significant difference between the overall accuracy of the VPM and LEFS in the area under the curve value (0.72 vs. 0.71) with respect to candidacy for surgery.

**Conclusions:**

This study provides substantial evidence towards the validity and reliability of the VPM outcome measure in patients with moderate to severe OA of the hip joint. Digitally based outcome measures have the potential of enhancing remote measurement of functional difficulties in specific situations.

**Clinical trial number:**

Not applicable.

## Introduction

Osteoarthritis (OA) is a highly prevalent joint disease associated with significant disability and increased use of resources. OA remains a major public health concern worldwide as the number of years of life lived with disability in older adults has increased exponentially over the past two decades [1, 2].

There are two distinct modes of outcome measurement in patients with OA. Patient-Reported Outcome Measures (PROMs) document patient’s physical difficulty using a questionnaire that addresses different aspects of a condition within a specific timeframe [3, 4]. Performance-based tests reflect a snapshot of an actual ability of a patient to execute a specific physical activity. Restrictions to in-person visits, initiated following the COVID pandemic ignited alternative modes of outcome measurement that incorporated remote assessment of functional difficulties. Despite resolution of pandemic-related restrictions, virtual care has remained an important part of routine care in the musculoskeletal field [5, 6].

The use of animated-figure videos for assessing activity limitations in patients with hip/knee OA was first introduced in 2014 [7–9]. The next generation innovative virtual assessment tool in the field of arthritis, the virtual performance measure (VPM) was introduced in 2023 and incorporated features of both self-report and performance based tools [10]. The term “virtual” indicates a task that is carried out, accessed, or stored by means of a computer, over a network as opposed to an actual physical presence of the patient and examiner. To be more relatable to elderly population, the VPM tool uses a human model with an appropriate age and more realistic facial expressions, which helps to reduce the imperfection and awkward body movement of an animated figure. In addition, the still presentation of an activity in a single image lacks different stages of a difficulty executing a task. Watching a video provokes an emotion in a person who suffers from disability while performing a task. A video of a moving person engages the patient in a more meaningful way [11]. The initial study of the VPM tool has shown promising results in patients with OA of the knee joint [10]. Considering the increasing utility of virtual care through digital technology, and different presentation of the lower extremity joints, assessment of accuracy of the VPM tool in patients with the hip joint OA is warranted.

The primary objective of this study was to establish reliability (internal consistency), and validity (factorial validity, convergent validity) of the VPM total score in patients with OA of the hip joint. The second objective was to compare the known-group validity of the VPM score with the traditional self-report and performance-based outcome tools in relation to candidacy for arthroplasty surgery, sex-related differences and use of an assistive walking device. We hypothesized that the VPM summated score would be reliable and valid in assessing limitations in physical activities secondary to OA of the hip joint. We expected that since the VPM incorporated both features of the self-report and performance-based tools, it would have a comparative known-group validity in relation to more traditional outcome measures.

## Methods

This was a cross-sectional study of patients seen at an academic tertiary care centre. Inclusion criteria included moderate to severe OA of the hip joint, which required a referral to a hip/knee Rapid Access Clinic (RAC), for consideration of joint replacement. These clinics accommodate patients who have not exhausted conservative treatment, or have failed non-surgical management and are a candidate for primary elective arthroplasty. Exclusion criteria included inability to read English, presence of cognitive conditions, lack of access to internet or inability to use online surveys. The study protocol was approved by the Human Ethics Research Board of the Sunnybrook Health Sciences Centre and all patients provided informed consent for participation in the study.

### Virtual performance measure

**Fig. 1:**
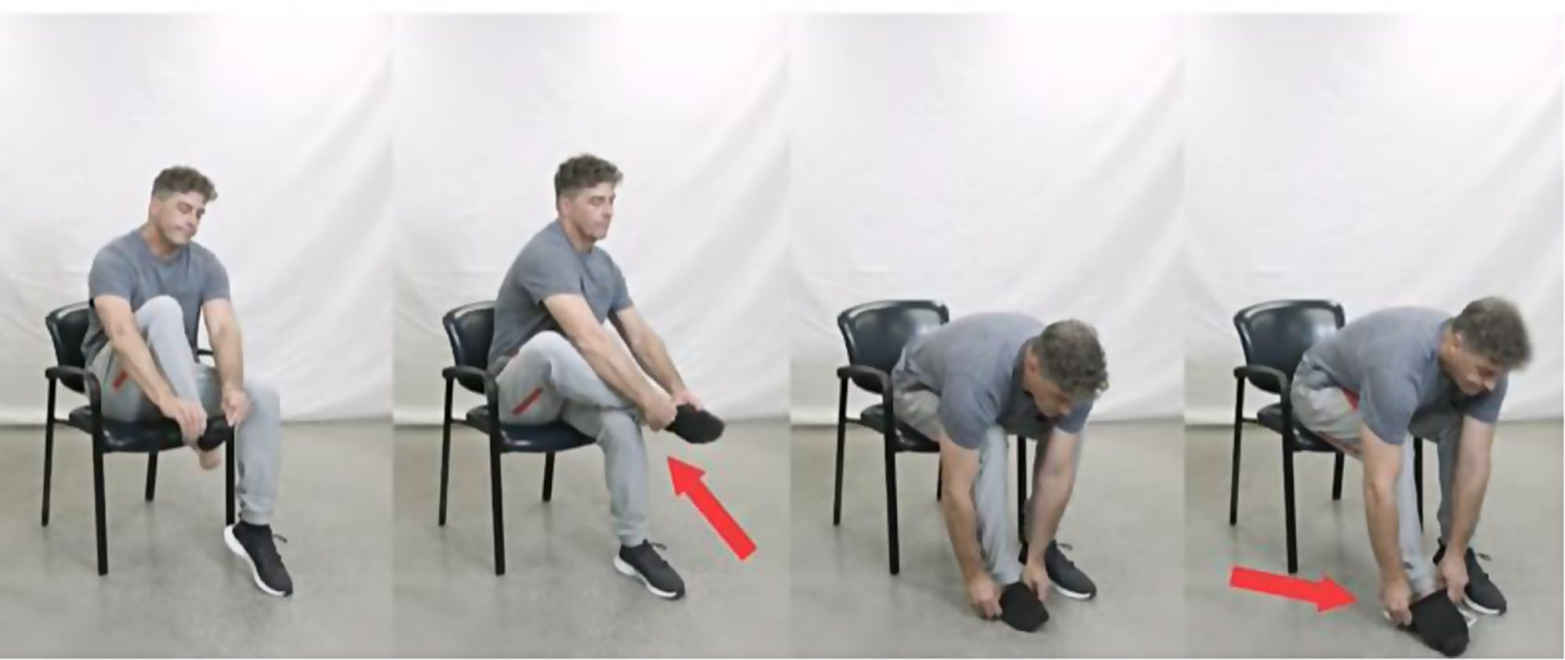
Putting on/taking off socks, with four levels of difficulty

**Fig. 2:**
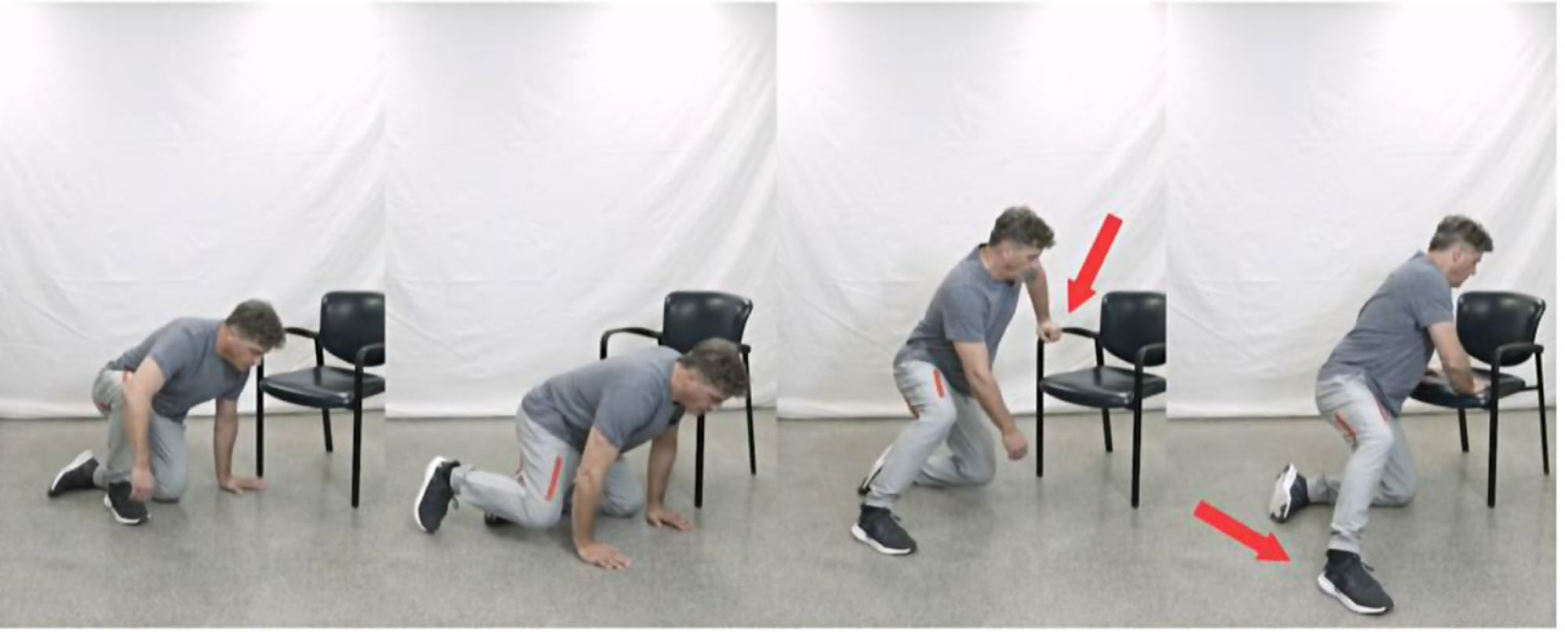
Sitting on the floor, with four levels of difficulty

Activities that were considered important to the OA population were selected based on input from physical therapists, occupational therapists, and patient representatives. The selected activities ranged from simple daily tasks (sitting on a chair) to more difficult activities (sitting /rising from the floor) to provide a wide range of difficulties. After multiple revisions, 40 videos with an age-appropriate model were chosen for 10 functional tasks. Previous video-based outcome measures have used a larger number of videos (17 items) [7]. We felt that 10 videos would be a good number for a straightforward score calculation while avoiding the burden of watching too many videos . The videos that encompassed the most affected daily activities by OA of the lower extremity included siting and rising from a chair, putting on/taking off socks, getting into the shower, walking a short distance on even ground, and ascending and descending stairs. More demanding activities such as picking up an item from the floor, sitting down and getting up from the floor were added to improve the variability of the tasks and avoid floor and ceiling effects of the total score [10]. Each functional task had four levels of difficulty (normal, mild, moderate, and severe) and an “unable to perform” option. The four videos were loaded simultaneously to improve efficiency of choosing the most accurate video. Patients could watch each task as many times as required using their smart phone, computer or tablet. The right leg was used as representing the affected leg and was labeled with a red tape. Arrows were used throughout the task to highlight subtle body adaptations for each activity (e.g. using the hand rest while sitting, or a reacher to put on socks). Patients were asked to choose the video that best reflected their own situation [Figs. 1 and 2].

### Clinical examination

Advanced practice providers (physiotherapist, occupational therapist) completed a standardized assessment tool; the osteoarthritis Severity Scoring System, commonly used scoring system by the advanced practice providers at the RACs in Ontario, Canada to determine appropriateness for surgical consultation. The severity score summarizes the results of clinical (history, physical examination, and pain intensity, ranging from 0 to 3), functional (self-report and performance measures, ranging from 0 to 3), and radiological examination (severity of osteoarthritis on imaging, scored from a rating of 0 to 2). The total severity score is calculated by summing all three scores, ranging between 0 and 8 with higher numbers indicating a higher severity. This scale has shown validity in patients with hip and knee arthritis [12, 13].

### Self-report outcome measures

The lower extremity functional score (LEFS) was used to assess reported functional difficulty [14] and P4 (4-item pain intensity measure) measured pain [15]. The LEFS has 20 questions, ranging from 0 to 80 with the highest numbers indicating better function. P4 documents pain at different times during the day (morning, afternoon, evening) and pain with activity, ranging from 0 to 40. LEFS and P4 have established reliability and validity in patients with lower extremity arthritis [2, 10, 14–18].

### Performance-based measures

Two performance-based tests were conducted: the 30- second Chair Stand Test (CST) [19], which document the maximum number of chair stand repetitions possible in a 30-second period and the 40-meter fast-paced walk (40 m FPWT) test respectively [20, 21]. These performance measures are reported to be valid and reliable in patients with hip/knee arthritis [10, 14–18].

The 30-sec CST and 40-m walk test were chosen as they are the most commonly used performance measures in the lower extremity population in North America and are routinely collected in rapid access clinics in Ontario, Canada. Similarly, the LEFS is a Canadian functional measure that has been validated many times in the OA population [2, 10, 14–18].

### Sample size justification and statistical analysis

Previous studies [7–9] that have used animated videos have shown correlation Coefficient of > 0.60 between the total score of animated physical activities and self-report and performance measures. With the Spearman Coefficient of 0.60, Fisher’s desired confidence interval (CI) of 0.30 and α = 0.5, a minimum of 86 patients were required [22]. The sample size of the factor analysis was based on prevalent rule-of-thumb of subject to item ratios of 10:1. We therefore, collected data on 100 patients with OA of the hip joint. The responses to 10 physical tasks represented in videos were added together and ranged between zero and 80. The score out of 80 was then transformed to a score out of 100 for an easier comparison, with 100 corresponding to normal function and zero representing complete disability. The details on calculating the total VPM score have been provided elsewhere [10].

### Reliability

As a measure of reliability, we examined the internal consistency of the single summed total score of the VPM, and each emerging factor, using Cronbach’s alpha coefficients. This analysis examined the pattern of association between different activities, with correlation of 0.70 being necessary for an acceptable alpha coefficient [23]. The test-retest reliability was investigated on patients with hip and knee OA in the original article [10] and was reported as 0.82 (*p* < 0.0001, CI _95%_: 0.75–0.90). This was not examined in the present study.

### Validity

Factorial validity examined the degree to which covariance among the 10 video responses resembled the covariation of underlying physical activities.

Cross-sectional convergent validity examined correlations between the VPM total score and traditional tools, the LEFS, P4, and actual performance measures, the 40 m FPWT and CST, and severity scoring system using Spearman’s correlations. We hypothesized a moderate correlation between the VPM total score and traditional outcomes as they use different modes of measurements. We expected a moderate reverse relationship between the VPM and the total severity score.

To examine the second objective, known group validity examined the ability of the VPM total score, LEFS, and performance-based measures in differentiating between surgical vs. non-surgical candidates for hip arthroplasty, men and women, and a need for use of a walking device using general linear model (GLM) analysis. It is expected that an outcome tool with a good known-group validity would be able to differentiate between different levels of the concept being measured. Candidates for immediate surgery and those who rely on walking aids are expected to suffer from higher levels of disability in performing daily tasks. In addition, the female sex is associated with higher levels of disability in the OA population [24–26]. We hypothesized that all measures would be comparable. The area under the curve (AUC) of the receiver operator characteristic curve assessed the overall predictive ability of the VPM and LEFS scores in relation to candidacy [27]. An AUC of above 0.70 is recommended for acceptable predictive validity [28].

### Ceiling and floor effects

Ceiling and floor effects (CFE) indicate the scale’s inability to discriminate at either extreme of the scale and refers to situations when a significant percentage of patients score the maximum or minimum score (e.g. 15%) [29]. A ceiling effect occurs when a high proportion of patients have maximum scores on the observed variable. The floor effect represent a high percentage of patients showing minimum scores on the variable. Statistical analysis was performed using SAS^®^ version 9.4 (SAS^®^ Institute, Cary, NC).

## Results

Table 1 shows the demographic characteristics of the sample included. Data of 100 patients, 64 (64%) females, 36 (36%) males, average age: 67 ± 10, min = 40, max = 92 years, with OA of the hip joint were examined. Of 100 patients, 81 were considered a candidate for surgical management, 18 were not considered an immediate candidate for arthroplasty and one patient was seen for a non-surgical opinion.

**Table 1:** Characteristics of sample included (*N* = 100)

Variables	Number (Percentage/SD)
**Age: Mean (SD)**	67 ± 10, min 40, max 92
**Sex: n (%)** • Female • Male	64 (64%) 36 (36%)
**Affected hip side: n (%)** • Left • Right • Bilateral involvement	40 (40%) 60 (60%) 22 (22%)
**Previous arthroplasty surgery: n (%)** • No • Knee joints • Opposite hip joint	76 (76%) 12 (12%) 12 (12%)
**Walking devices: n (%)** • Yes • No	25 (25%) 75 (75%)
**Gait: n (%)** • Normal • Abnormal	17(17%) 83(83%)
**Range of motion examination: mean (SD)** • Flexion • Abduction • Adduction • External rotation • Internal rotation	94 (14) 25 (11) 15 (11) 27 (16) 7 (12)
**Severity Score: mean(SD)** • Clinical (0–3) • Functional (0–3) • Radiological (0–2) • Total score (0–8)	2.05 (0.54) 1.84 (0.61) 1.71 (0.48) 5.60 (1.3)
**Outcome measures** • LEFS • P4 • Chair Sit Test • 40 m fast paced walk	32 (16) 22 (9) 11 (4) 33 (9)
**Proceeding to surgeon consult** • No • Yes, Candidate for arthroplasty • Yes, Second opinion	18 (18%) 81 (82%) 1 (1%)

SD: Standard Deviation

LEFS: Lower extremity functional score

P4: 4-item pain intensity measure

In terms of reliability, the Cronbach’s alpha coefficient was 0.88, indicating good reliability [23].

The Cronbach’s alpha coefficients were 0.86 and 0.79 for factor1 and 2 respectively. Factor analysis of 10 physical tasks showed two distinct domains (Table 2). The first domain included activities that required weight-bearing flexion of the hip joint while moving forward, (ascending and descending stairs, and getting into the shower) and activities that required loaded hip flexion, adduction and rotation (sitting and rising from the floor). The second domain involved hip flexion while balancing/stabilizing the upper body (sitting down/rising from chair), walking on a flat surface, picking up an item from the floor, and putting on/taking off socks. There was no cross loading of the activities at 0.5 significance level, indicating that the factors were distinct and represented separate concepts. Overall, the variance warranted separate interpretation of ten activities, but the aggregated score formed a coherent dimension.

**Table 2:** Factor analysis: Promax oblique rotation. Factor analysis examined the degree to which covariance among the responses resembled the relationship between physical tasks

	Factor1	Factor2
Sitting down	.	0.91420
Rising from chair	.	0.89674
Picking from floor	.	0.50125
Walking on flat surface	.	0.55106
Putting on/taking off socks		0.53698
Descending stairs	0.71823	.
Ascending stairs	0.72481	.
Rising from floor	0.89113	.
Siting on the floor	0.76421	.
Getting into the shower	0.65210	.

In terms of cross-sectional convergent validity, the correlations between the VPM total score and the self-report and one performance measure were moderate: LEFS (*r* = 0.68, *p* < 0.0001), P4 (*r* = 0.52, *p* < 0.0001) and the 40 m FPWT (*r* = 0.55, *p* < 0.0001). The correlation between the VPM and CST was low (*r* = 0.31, *p* = 0.01). In terms of severity score that incorporated clinical, functional, and radiological assessment, the correlation was highest for the functional abilities (*r*=- 0.63, *p* < 0.0001) and lowest for radiological findings (*r*=-0.12 *p* = 0.26). The total severity score correlated with the VPM score at − 0.52, *p* < 0.0001.

**Fig. 3:**
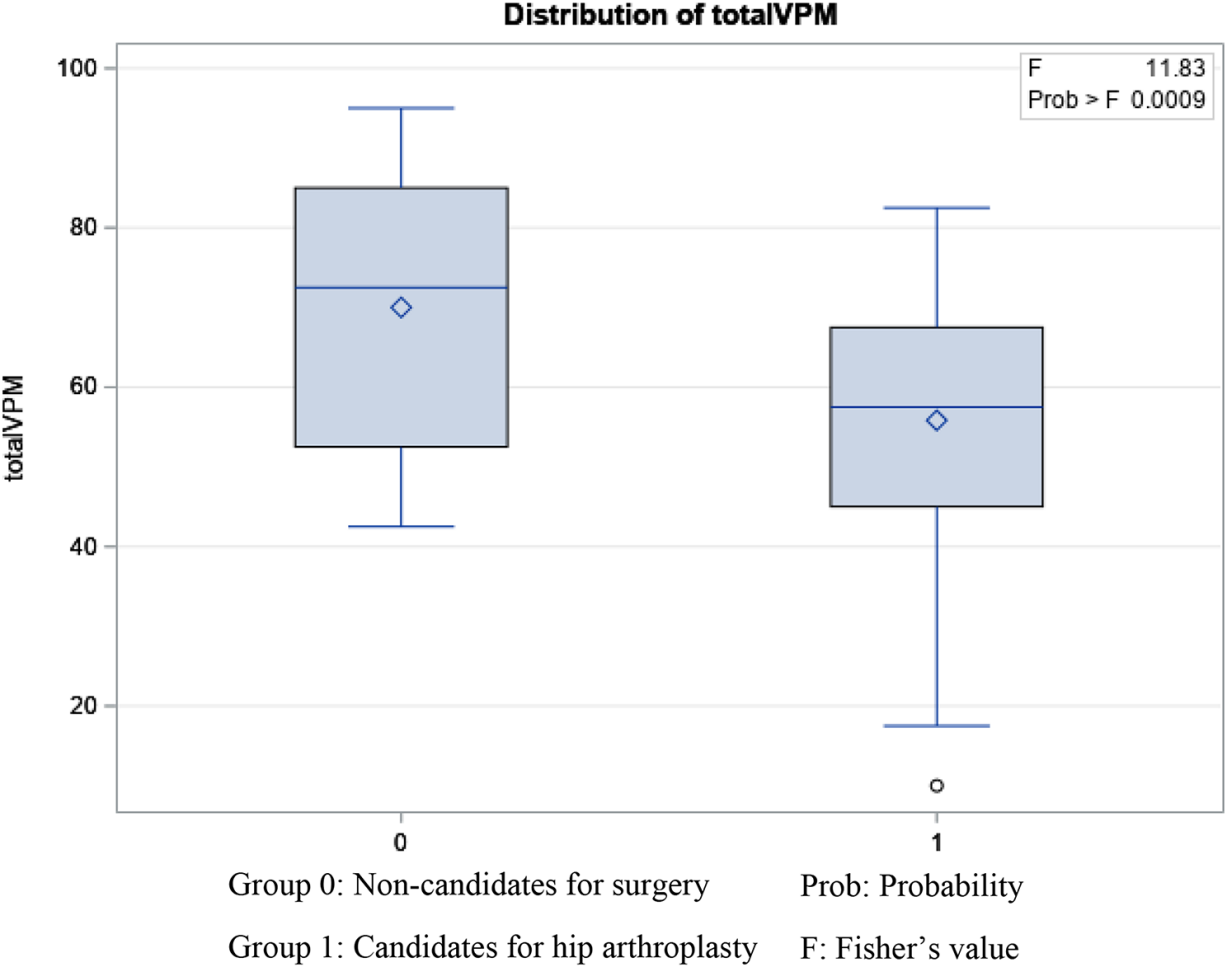
Distribution of virtual performance measure total score between candidates and non-candidates for knee arthroplasty

**Fig. 4:**
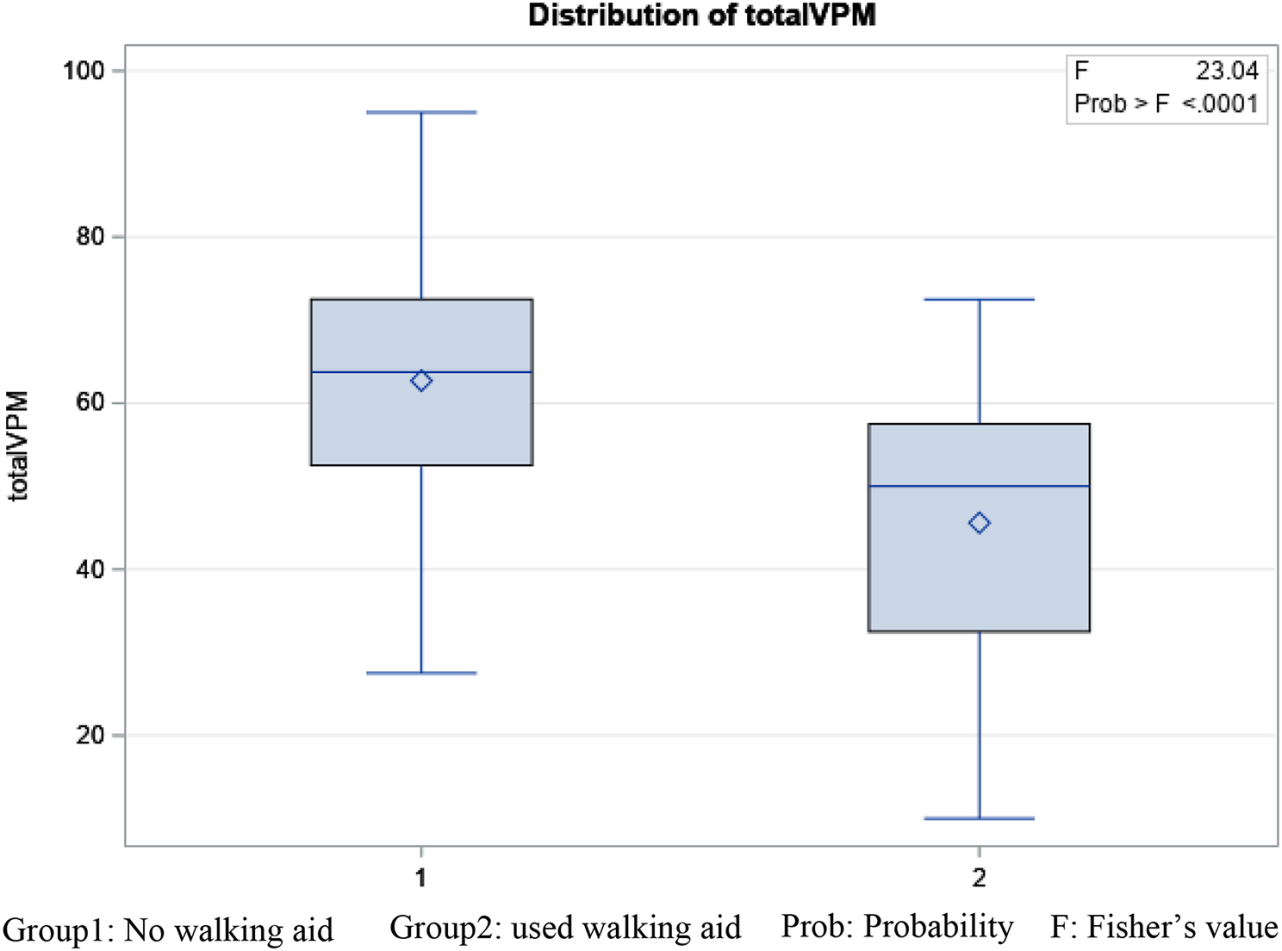
Distribution of virtual performance measure total score based on use of walking aids

In terms of known-group validity, our second objective, the total VPM was able to differentiate between candidates for hip arthroplasty vs. non-candidates (F = 11.83, *p* = 0.0009) [Fig. 3], those who required walking aids vs. those who did not use any assistive devices (F = 23.04, *p* < 0.0001) [Fig. 4], and men and women (F = 4.24, *p* = 0.042) in favor of men. The factor of age did not affect the VPM total score (F = 0.01, *p* = 0.99) in this sample. Similarly, the LEFS showed ability to differentiate between arthroplasty candidates (F = 13.38, *p* = 0.0004), and those who required walking aids (F = 11.41, *p* = 0.001). LEFS did not show a statistically significant difference between men and women (*P* > 0.05) in the sample studied. The performance measures both differentiated between men and women (CST: 2.15, *p* = 0.030, 40 m test: 2.14, *p* = 0.027). The scores of both measures, however were similar in candidates vs. non-candidates for surgery (*p* > 0.05) and those who used a walking device vs. those who did not (*p* > 0.05).

The logistic regression that calculated the AUC values for the overall differentiation between candidates vs. non candidates showed an AUC of 0.72 for the total VPM and 0.71 for LEFS indicating acceptable predictive validity (both tools were able to accurately differentiate between candidates and non-candidates above 70% of the time). There was no statistically significant difference between two outcome measures. The VPM total scores varied from 10 to 95 and there was no floor or ceiling effects.

## Discussion

Virtual tools facilitate access and assessment of patients without actual presence of the patient in the clinic or hospital in unfavorable situations. Considering the evolution of digital data in health care system, examination of validity and reliability of newly developed digital tools is vital to ensure accuracy of clinical information.

In the present study, the VPM total score demonstrated high internal consistency reliability with good convergent and known-group validity in patients with OA of the hip joint. Factorial validity identified two separate dimensions that represented different physical demands on an arthritic hip joint. The results of the present study is consistent with the results of the original study on patients with OA of the knee joint in most aspects except for the factorial validity [10]. The internal consistency of the VPM in the hip OA was 0.88 vs. 0.90 in the knee sample, which is not clinically significant and may just show a higher correlation among functional items in knee OA.

The convergent validity between the VPM and LEFS, P4, and 40 m FPWT was moderate. The knee sample showed a correlation of *r* = 0.60 and 0.46 with the CST and 40 m FPWT respectively. In the hip sample, the correlation was higher with the 40 m FPWT (*r* = 0.55) and insignificant with the CST (*r* = 0.31). This is consistent with a study by Kennedy et al. [24] who compared patients with knee OA with hip OA. The authors found that patients with hip OA were significantly more disabled than patients with knee OA in self-paced walk. There is a possibility that since knee extensors contract eccentrically during the lowering phase of CST, this activity correlates with function more linearly in knee OA than Hip OA. The OA of the hip joint is associated with reduced gluteal muscle strength, which may affect walking more significantly. Considering neither actual performance score could differentiate between surgical candidates and non-candidate, the clinical significance of this finding is debatable.

In the previously conducted study that involved patients with knee OA [10], three distinct domains represented three levels of complexity of functional tasks. In the present study that involved hip OA, there was less variability in the number of domains (two factors). This may be explained by biomechanical changes due to osteoarthritis, which has a differential impact on the overall stability, coordination, axial loading, and strength of the leg muscles depending on the site of joint involvement. One other possible explanation is that the severity of OA in the present study was higher (80% were candidates for hip arthroplasty vs. 60% for knee arthroplasty) than the original study [10]. Osteoarthritis leads to capsular and joint structure pathologies, affecting muscle strength, postural stability, neuromuscular control, and joint proprioception [30, 31]. Studies performed on women with hip OA have shown significant disturbance in proprioception and posture as compared with the healthy controls [32]. In terms of theoretical constructs of physical function found in the present study, sitting and getting up from a chair and putting on/taking off socks fell in the same factor. Both of these tasks require about 90º of hip flexion for a comfortable execution, with the latter activity requiring a functional range of external rotation as well. Considering the hip OA affects internal rotation and extension more than flexion and external rotation [33], better external rotation appears to be more closely related with less demanding functional activities such as siting/getting up from a chair and walking on a level surface. The limitation in external rotation angle is reported to correlate with postoperative outcomes and patient satisfaction for basic tasks such as putting on/taking off socks and is consistent with our results [34]. The more physically demanding tasks such as sitting and rising from floor and ascending/descending stairs and getting into the tub involve a loaded flexion, hip extension and complex rotation with good muscular strength and fell in a more demanding task domain.

There is minimal information on direct comparison between physical demands on hip and knee joints. Samuel and colleagues [35] examined the physical performance of healthy older adults in terms of functional demands encountered at the hip and knee joint. The authors reported that stair navigation was the most demanding activity in healthy elderly. Overall, the biomechanical and joint-specific OA-related changes may explain some of the differences in factor validity of the functional limitations between the hip and knee joint OA patients. While the level of difficulty in each task depends on multiple factors, such as severity of the pathology, general medical health, age, presence of obesity and other comorbidity factors, it appears that stair navigation and sitting and raising from floor remain the most challenging activities in both samples. The consistent finding between hip and knee OA was lack of linear relationship between function and severity of the radiological findings, a well-known fact in the field of arthritis [36, 37].

Using a biopsychosocial approach in chronic pain and arthritis is becoming more common as it help clinicians and researchers to improve the accuracy of their measurement and management. Recent studies have found a discordance between self-report and actual performance in the OA population with the fear-avoidance and positive affect contributing to this mismatch [38, 39]. Hybrid outcome tools have a potential in reducing this gap and their value needs to be properly assessed in future studies.

The VPM has many potentially valuable clinical uses. The VPM can be used as a patient reported outcome measurement to determine improvements following physiotherapy as well as surgical intervention. It can also be used to assist in decision making for suitability and timing of surgical intervention. The VPM would facilitate virtual assessments by gathering information on patient’s functional level as well as supporting in-person visits by having patients complete the VPM ahead of time to maximize the in-person visit. The VPM can also be a valuable way that patients can track their progress. Patients can look back after physiotherapy or surgery and note their function prior to the intervention to see how much progress they have made.

In summary, the results of the present study adds to the body of literature in the field of virtual care and digital outcome measurement with a potential to transform health care and improve clinical staff and patient’s experience. With the continuous use of computers by older individuals, virtual outcomes could lessen the burden of travelling for patients and their families and reducing the time-consuming one-on-one measurements, while improving the overall efficiency of arthritis care in patients with hip OA both before and after surgery.

### Limitations

This study examined the reliability and validity of the VPM in patients diagnosed with severe arthritis of the hip joint seen at a tertiary care centre, of whom about 80% were surgical candidates. This high level of OA severity may affect the generalizability of our findings to community clinics with a less prevalence of severe pathology. Future studies should examine the applicability of this innovative tool in more diverse populations. The test-retest reliability will need to be examined in different populations as well. Use of other statistical approaches, such as Rasch analysis may further provide a detailed mapping of item difficulty across the functional spectrum, identify potential gaps or redundancies, and optimize the scoring of the VPM with a greater precision and applicability in assessing functional difficulties. Role of obesity on accuracy of the tool was not examined in this study and needs to be investigated. In addition, longitudinal studies are required to examine the longitudinal validity of the VPM for making decisions at the individual patient level.

## Conclusions

This study provides substantial evidence towards the validity and reliability of the VPM outcome measure in patients with moderate to severe OA of the hip joint. Digitally based outcome measures have the potential of enhancing remote measurement of functional difficulties in specific situations.

## Data Availability

Due to ongoing nature of the study, partial data are available from the corresponding author on reasonable request.
